# Integrating medical physics into an EMR‐based radiology feedback system for quality improvement

**DOI:** 10.1002/acm2.70227

**Published:** 2025-09-09

**Authors:** Megan K. Russ, Justin Solomon, Steve Bache, Nicole M. Lafata, Erin B. Macdonald, Ehsan Samei

**Affiliations:** ^1^ Clinical Imaging Physics Group Duke University Health System Durham North Carolina USA; ^2^ Department of Radiology School of Medicine Duke University Durham North Carolina USA; ^3^ Center for Virtual Imaging Trials Duke University Durham North Carolina USA

**Keywords:** continuous improvement, feedback communication systems, quality feedback

## Abstract

**Introduction:**

Medical physicists play a critical role in ensuring image quality and patient safety, but their routine evaluations are limited in scope and frequency compared to the breadth of clinical imaging practices. An electronic radiologist feedback system can augment medical physics oversight for quality improvement. This work presents a novel quality feedback system integrated into the Epic electronic medical record (EMR) at a university hospital system, designed to facilitate feedback from radiologists to medical physicists and technologist leaders.

**Methods:**

The feedback system was designed to enable radiologists to report quality issues directly through a streamlined survey during report dictation. The feedback encompasses technical details including image noise, artifact, and contrast issues, as well as acquisition‐related concerns such as positioning errors or protocol deviations. Submissions are routed to modality‐specific teams consisting of technologist leaders and medical physicists, who investigate and address reported issues. The roles of medical physicists in this feedback system were evaluated over a 31‐month period.

**Results:**

Physicists addressed 9.3% of 515 tickets that warranted follow‐up, with greater involvement in resolving technical quality issues including artifacts and issues related to noise and image contrast. Examples of physicist‐led interventions included correcting radiography image processing settings, optimizing computed tomography dose settings, and identifying trends in ultrasound quality issues that prompted protocol updates and staff training.

**Conclusion:**

This work demonstrates the value of radiology quality feedback systems and the opportunity to address issues not typically identified during routine medical physics quality assurance. By leveraging radiologist feedback, physicists can enhance clinical practice, promote continuous improvement, and ensure consistent, high‐quality imaging and safety for patients.

## 1 INTRODUCTION

Medical physicists play a critical role in ensuring clinical image quality and patient safety. Part of a physicist's role in ensuring image quality and patient safety includes performing routine equipment and protocol evaluations. These routine evaluations may be limited in both frequency and scope compared to the breadth of clinical applications due to practical constraints such as time and challenges in simulating patients. As a result, routine evaluations may not capture the full range of issues that can be identified and remedied by physicists. For example, annual quality assurance tests provide a comprehensive evaluation of hardware performance and integrity, baseline image quality, and patient safety, but may only evaluate the image quality in a few clinical protocols and has a sampling rate of only once per year. More frequent quality control (QC) methods are likewise equipment based and of limited frequency. Annual evaluations may be augmented by patient image‐based monitoring systems that can detect changes in dose and image quality metrics, but these tools are not currently available for all imaging modalities and may be limited in what exam types these tools are developed for.^1^, ^2^, ^3^ Accessing feedback from radiologists, who have a high sampling rate of patient images and can discern when artifacts or image quality variations represent a risk to diagnostic quality, is the logical next step for medical physicists in working towards continuous quality improvement.

Communication systems aimed at facilitating feedback for quality improvement purposes between radiologists and technologists have previously been created and implemented in radiology departments. Kruskal et al. developed a web‐based tool for communicating a broad list of quality issues relating to image acquisition, equipment, technologist, and staff issues that spurred the creation of quality assurance projects and additional staff training.^4^ Ong et al. presented a streamlined quality reporting and tracking solution directly linked to their hospital's picture archiving and communication system (PACS) that facilitated communication between technologists and radiologists, allowing for identification of staff requiring additional training to meet performance standards.^5^ Goldberg‐Stein et al. created a quality improvement tool that was integrated into their radiologist workflow through their hospital's electronic medical record software, which increased radiologist engagement.^6^ Each of these solutions demonstrate the value of a feedback system in creating opportunities for quality improvement within radiology departments; however, none of these communication systems explicitly state the inclusion of medical physicists as participants in their communication systems, or describe any contributions they may have made to quality improvement efforts based on feedback received from radiologists.

We designed and deployed a new radiology quality feedback system in the electronic medical record software, Epic (Epic Systems Corporation, Verona, Wisconsin, USA), for a university hospital system. This feedback system differs from previously reported quality feedback tools in that physicists were intimately involved in its design and are included as recipients of feedback from radiologists, alongside their technologist leader colleagues. The purpose of this work was to evaluate the roles that medical physicists took on within the feedback system, and the added value that resulted from medical physicists addressing issues raised by radiologists.

## 2 METHODS

Similar to the system created by Goldberg‐Stein et al., this communication system was built into the hospital system's electronic medical record, Epic, a foundational component of radiologists’ workflow. Epic has built‐in messaging tools that can integrate into the electronic medical record of patients whose exams were the subject of radiologist feedback. This feedback system provides radiologists with access to an optional short survey form during report dictation where they can provide constructive feedback on one or more specific aspects of an exam's quality without their feedback being recorded in the patient's record.

**FIGURE 1:**
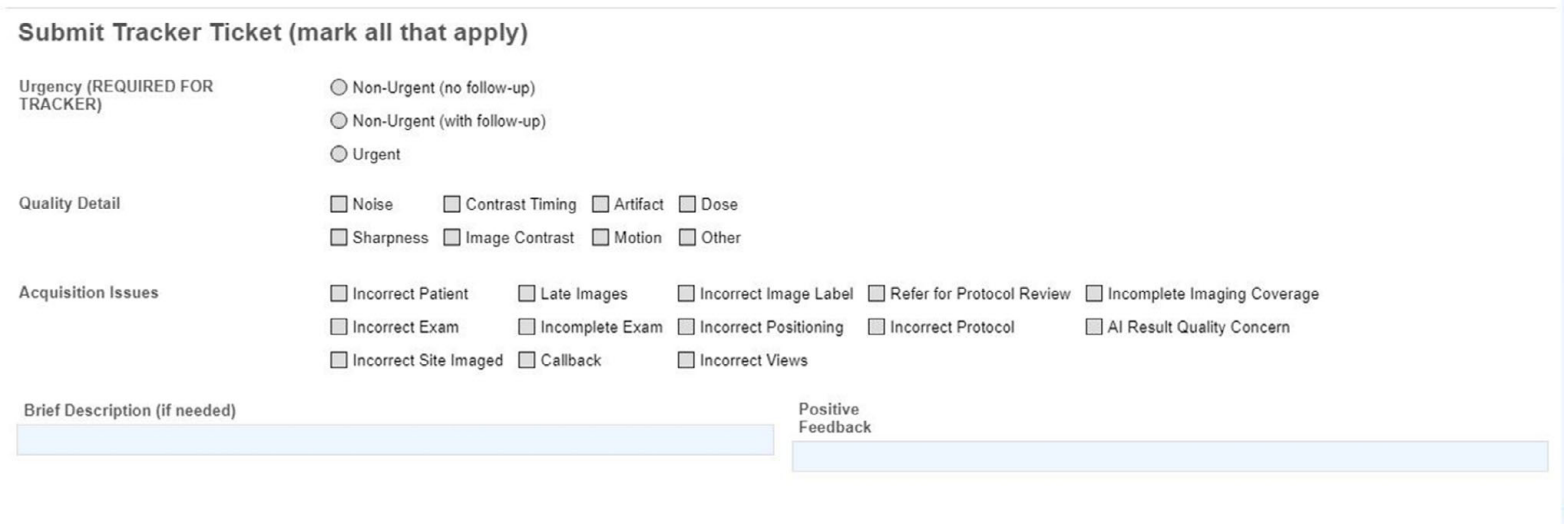
An example of the radiologist feedback form. The feedback form used by radiologists, showing the urgency scale, all check boxes, and free text response boxes.

An example of the survey form is shown in Figure 1. Two categories of check boxes were available to radiologists on the survey form to expedite feedback entry: quality detail and acquisition issues. The quality detail boxes included noise, contrast timing, artifact, dose, sharpness, image contrast, motion, and other. The acquisition issues boxes included incorrect patient, late images, incorrect image label, refer for protocol review, incorrect exam, incomplete exam, incorrect positioning, incorrect protocol, incorrect site imaged, callback, incorrect views, and incomplete imaging coverage. Radiologists were able to select multiple checkboxes on each ticket. Two text boxes were available for free form text entry. One of the textboxes was for a brief description of an issue, the other was specifically used for positive feedback. This design was informed by an earlier version of the feedback system at our institution that identified the most common quality feedback categories.

Once the survey form is filled out and the radiologist has signed and saved their report, a message is generated and sent to an inbox accessible to the individuals involved in oversight of this feedback system. These individuals, both medical physicists and technologists, review all feedback received in the inbox and route it to the correct modality group. Each modality group consisted of technologist leaders and medical physicists who supported that modality at all sites within the hospital system. Radiologists were able to request a follow‐up message from modality group members using a three‐point ticket urgency scale, which can be seen in Figure 1. Individual modality group members were able to claim tickets relevant to their site or practice at their discretion. Depending on the issue raised in the radiologist's feedback, action taken to investigate and resolve the issue at hand may have involved collaboration between multiple group members, or action by one individual. Final follow‐up messages to the radiologists included an explanation after investigation of what led to an issue occurring and/or a description of action taken to mitigate the issue for future studies. The person who routed the ticket to the modality group received a copy of follow‐up messages if the responder indicated “Reply All” on their response.

To assess the added value of including medical physicists in the group of recipients, the number and the topic of tickets physicists responded to were compared against those technologist leaders responded to. Follow‐up messages from the period of March 28, 2022, to October 31, 2024, in the inbox owned by the medical physics group were examined for the role of the responder and the topic of the ticket. The ticket topic was assessed using the checkboxes selected on each ticket. For tickets that only had a free text response and did not have a checkbox selected, the most suitable checkbox based on free text content was selected by the authors. The role of the ticket responder (i.e., medical physicist or technologist leader) was identified by information available in Epic messaging tools.

In addition to the accounting performed of tickets, three anecdotes are also presented, showcasing examples of meaningful action medical physicists took in response to radiologist feedback through this system.

## 3 RESULTS

During the period studied, there were 515 follow‐up messages from technologist leaders and medical physicists collected in the inbox owned by the medical physics group. For comparison, during the same period, there were 1370 tickets submitted by radiologists with an urgency designation indicating a follow‐up message was desired. Medical physicists responded to 9.3% of these tickets and technologists responded to the other 90.7%.

**FIGURE 2:**
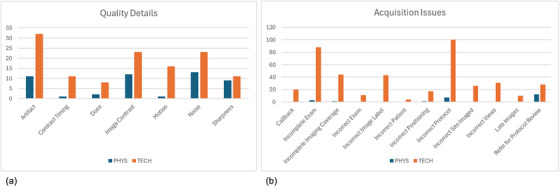
Topic breakdown of tickets medical physicists and technologists responded to. Bar graph demonstrating the number of tickets responded to by medical physicists (PHYS) and technologist leaders (TECH) for each check box in the quality details (a) and acquisition issues (b) categories.

Figure 2a and b show the number of tickets that physicists and technologists (labeled as PHYS and TECH in Figure 2a and b, respectively) responded to for each check box in the quality detail or acquisition issue categories. There were 446 instances of acquisition issue checkboxes being selected, and 173 instances of quality detail checkboxes selected. Medical physicists responded to 28.3% of quality detail‐focused tickets, and 5.8% of acquisition issue‐focused tickets.

Three anecdotes are presented below detailing the actions physicists took as a result of being alerted to issues through this feedback system.

## 4 EXAMPLE 1: RADIOGRAPHY IMAGE QUALITY

A ticket was submitted that contained the free‐text feedback “markedly limited by excessive image contrast. Lungs basically not assessed” for an anteroposterior (AP) chest x‐ray exam. This chest x‐ray exam required use of the free detector, as the patient was seated in a wheelchair and unable to stand at the wall Bucky. The team of x‐ray physicists investigated the issue further, and found there had been multiple instances of excessive image contrast in AP chest x‐rays performed with the free detector in the same room.

**FIGURE 3:**
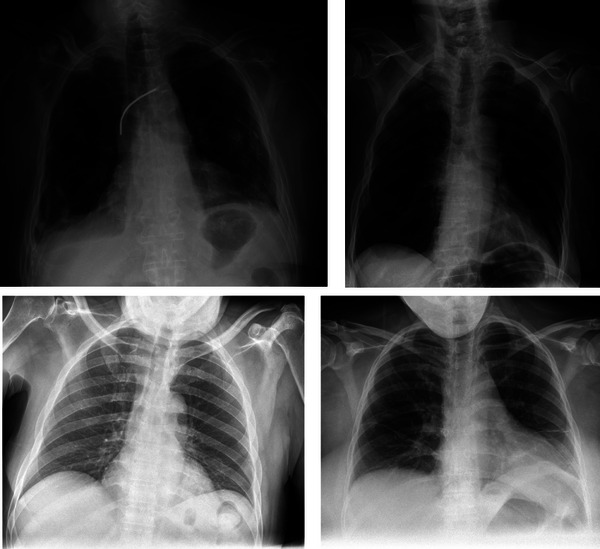
Chest radiographs demonstrating improvement in contrast following physicist intervention. Images demonstrate the difference in image appearance when using the incorrect grayscale ranger (top row), and the improvement in lung detail after updating the protocol to use the lung range selection (bottom row).

The root cause of this was identified as the processing algorithm selecting incorrect anatomy as the region to optimize for grayscale range mode. The x‐ray physicists found that, for patients imaged using the free detector for AP chest exams, updating the ranging mode to select the correct anatomical region improved the appearance of contrast in the lungs to a level approved by radiologists. Physicists updated the ranging mode in the image processing protocol for AP chest views, and the issue has not been encountered since. Figure 3 shows example patient images acquired using the incorrect grayscale ranger, and the improvement in contrast seen after using the lung range selection.

## 5 EXAMPLE 2: COMPUTED TOMOGRAPHY IMAGE QUALITY

**FIGURE 4:**
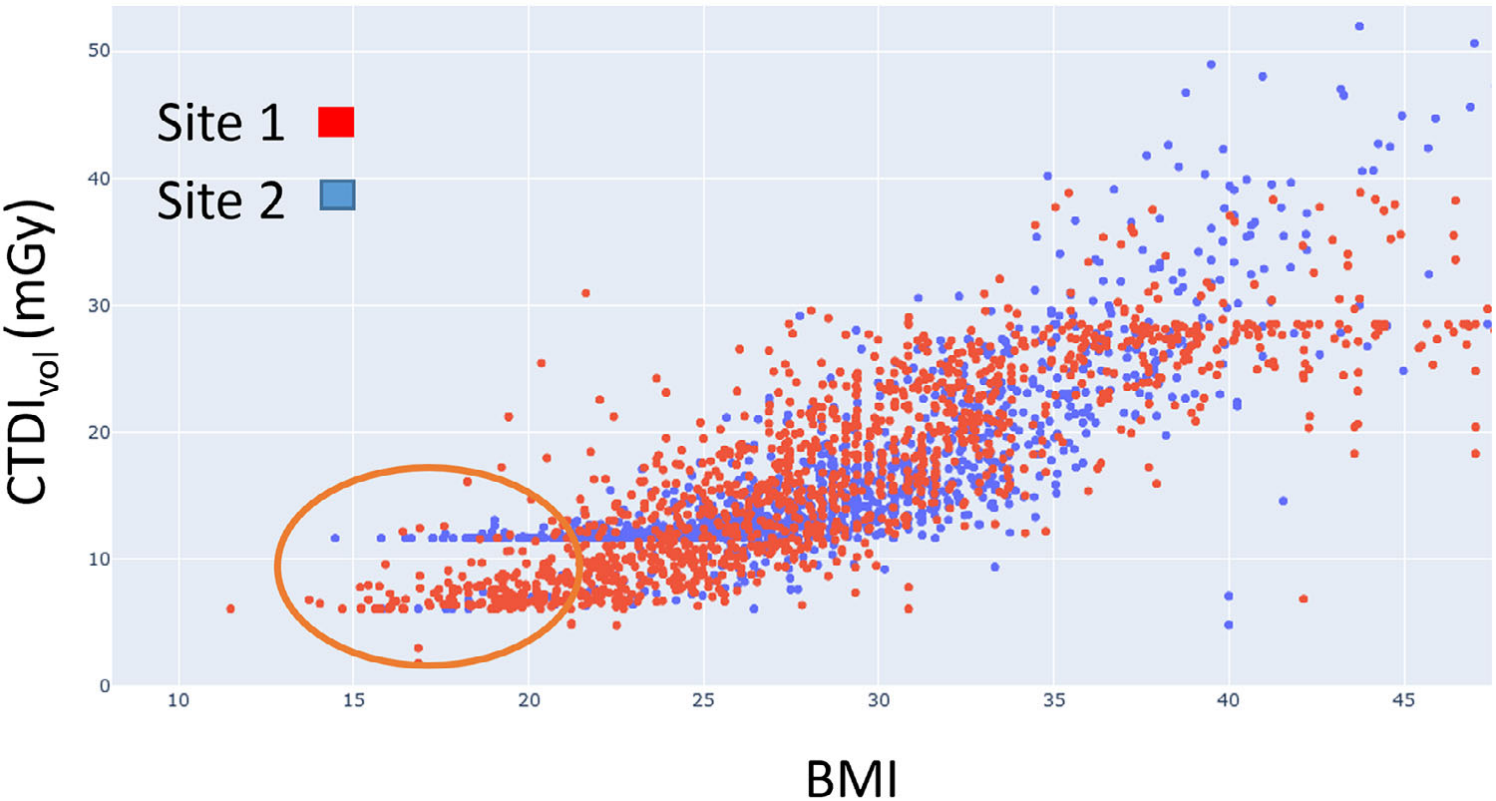
CTDIvol data as a function of BMI for Sites 1 and 2. The CTDIvol data for Sites 1 and 2. Circled region exhibits the lower dose floor for patients with low BMI as a result of the reduced pitch in Site 1′s scan protocol.

Three tickets were submitted regarding noisy chest/abdomen/pelvis (CAP) and abdomen/pelvis (AP) exams at one of the hospitals in our hospital system, identified as Site 1. In each case, the patient was thin, and their arms were up, so the noise level in their images was noteworthy. The scan protocols for CAP and AP exams at Site 1 were compared by computed tomography (CT) physicists to the CAP and AP protocols for the same scanner manufacturer at a different hospital, identified as Site 2, within the hospital system. They determined that the lower limit tube current at Site 1 matched the lower limit tube current at the reference hospital, but the pitch had been adjusted from ∼0.5 to ∼1.0 to speed up scan times, effectively halving the dose floor. The impact of this difference in dose floor was reflected in the volume CT dose index (CTDIvol) data plotted for both sites as a function of body mass index (BMI) (shown in Figure 4). The issue of image noise was resolved by adjusting the lower limit tube current at Site 1 to match the noise level of Site 2 without adjusting the pitch. The question of whether to standardize the pitch across these two hospitals was addressed at a subsequent CT protocol review meeting.

## 6 EXAMPLE 3: ULTRASOUND TICKET TREND ANALYSIS

Ultrasound (US) received fewer tickets per week on average than radiography, CT, and magnetic resonance imaging (MRI), and the vast majority of submitted tickets were topics that were handled by US sonographer leadership. After this feedback system was active for nearly a year, all ticket data was compiled and analyzed for trends by physicists overseeing the feedback system. That analysis revealed that 59% of US tickets pertained to incomplete exams due to missing views and/or missing measurements, and an additional 10% of tickets concerned incorrect image labels. These findings precipitated discussions with radiologists and sonographer leadership to identify the root cause of the problem and led to formulating a plan to ensure greater consistency in patient US exams. This involved a revision of the internal protocol documents for common study protocols that were out of date, a site‐wide survey performed by US physicists of the protocols built on US scanners to ensure compliance with updated protocol documents and cross‐fleet consistency, as well as offering education to sonographers unfamiliar with internal protocol documents or scanner protocol and measurement tools.

## 7 DISCUSSION

A quality feedback system was built and deployed in the electronic medical record software, Epic, for a university hospital system. This feedback system differed from previously reported quality feedback tools by including medical physicists in the list of recipients of radiologist feedback, along with their technologist leader colleagues. This work demonstrated the added value of medical physicists in radiology feedback systems by expanding the contribution of physicists for improved imaging care.

While most feedback from radiologists in the period studied pertained to acquisition issues and was handled by technologists, evidenced by the greater frequency of acquisition issue checkboxes selected in the response messages studied, physicists handled a substantial fraction of the technical quality detail‐focused tickets, most commonly tickets related to artifacts, noise, and image contrast. Addressing questions on these topics is well within a medical physicist's expertise, and while technologists have a sound understanding of the factors influencing these image characteristics, a physicist will have the ability to discern the issues more systematically, whether an issue is likely to affect other patient cases, whether troubleshooting by a physicist should be performed, and whether service of a system is required for resolution.

While reviewing the follow‐up messages for this work, multiple messages were noted where the responses from technologist leaders indicated that they were reaching out to the medical physicist who supported their modality to involve them in resolution of an issue raised by a radiologist. This alone serves to demonstrate the value that physicists have in this feedback system, that technologist leaders can readily loop in their physicist colleagues and lean on them for issues relevant to their expertise.

Radiologist response to the feedback system has been widely positive, as reflected in their consistent use of the tool to share both concerns and commendations. Radiologists, technologists, and medical physicists have all played a key role in refining the system, offering suggestions to streamline the ticket submission process through auto hotkeys, reduce messaging redundancy by customizing recipient lists for tickets where no follow‐up is requested, and enhance data visualization through improved reporting formats for trend analysis. This ongoing engagement underscores a shared commitment to continuous quality improvement through communication and collaboration. The anecdotes presented detail the depth at which physicists can investigate the root cause of issues raised in submitted tickets, both at the individual ticket level and through ensemble analysis. The radiography and CT anecdotes both demonstrate the increased breadth of clinical protocols examined and potentially improved through access to radiologist feedback, beyond the standard protocols covered during routine quality assurance procedures. These examples cited are just a few pulled from many cases of medical physicist engagement in quality improvement efforts initiated through inclusion in this communication feedback system. The increasing ubiquity of Epic as hospital electronic medical records systems means that tools like these presented could be implemented elsewhere and improve the quality of radiology practice at hospitals worldwide.

Some messages were excluded from this analysis. First, responses to tickets with only positive feedback were excluded. Additionally, only tickets pertaining to CT, MR, radiography, and US were included in this analysis. Though there are recipient groups for fluoroscopy, mammography, and nuclear medicine, these modalities are still being integrated into our clinical process. Other institutional feedback mechanisms were in place for these modalities at the time of the feedback system launch.

## 8 CONCLUSION

This feedback tool integrated into radiologists’ workflow through Epic is a step towards continuous improvement in patient care by creating an open line of communication between radiologists, technologists, and medical physicists. Including physicists in this feedback system has led to meaningful improvements in patient care at our institution for issues they may not have been aware of without access to radiologist feedback.

## AUTHOR CONTRIBUTIONS

Megan K. Russ conceived the study, designed the comparison, collected and analyzed the data, and wrote the manuscript. Justin Solomon contributed to the design of the analysis and contributed significantly to the manuscript's revision. Steve Bache contributed the CT anecdote and contributed to the manuscript's revision. Nicole M. Lafata and Erin B. Macdonald contributed the radiography anecdote and contributed to the manuscript's revision. Ehsan Samei guided the project's conceptual design and contributed to the manuscript's revision. All authors reviewed and approved the final version.

## CONFLICT OF INTEREST STATEMENT

The authors have no relevant conflicts of interest to disclose.

## References

[1] Lin Y , Luo H , Dobbins JT , et al. An image‐based technique to assess the perceptual quality of clinical chest radiographs. Med Phys. 2012;39(11):7019‐7031. doi:10.1118/1.4760886 23127093

[2] Christianson O , Winslow J , Frush DP , Samei E . Automated technique to measure noise in clinical CT examinations. AJR Am J Roentgenol. 2015;205(1):W93‐W99. doi:10.2214/AJR.14.13613 26102424

[3] Sanders J , Hurwitz L , Samei E . Patient‐specific quantification of image quality: an automated method for measuring spatial resolution in clinical CT images. Med Phys. 2016;43(10):5330. doi:10.1118/1.4961984 27782718

[4] Kruskal JB , Yam CS , Sosna J , Hallett DT , Milliman YJ , Kressel HY . Implementation of online radiology quality assurance reporting system for performance improvement: initial evaluation. Radiology. 2006;241(2):518‐527. doi:10.1148/radiol.2412051400 17057072

[5] Ong L , Elnajjar P , Nyman CG , Mair T , Juluru K . Implementation of a point‐of‐care radiologist‐technologist communication tool in a quality assurance program. AJR Am J Roentgenol. 2017;209(1):W18‐W25. doi:10.2214/AJR.16.17517 28402126 PMC5565840

[6] Goldberg‐Stein S , Kaplun O , Scheinfeld MH , Burns J , Miller T , Erdfarb A . Making feedback easy: a workflow‐integrated quality improvement tool increases radiologist engagement in the technical quality of imaging examinations. J Am Coll Radiol. 2018;15(10):1443‐1447. doi:10.1016/j.jacr.2018.03.030 29724624

